# Site-specific endometrial injury improves implantation and pregnancy in patients with repeated implantation failures

**DOI:** 10.1186/1477-7827-9-140

**Published:** 2011-10-21

**Authors:** Shang Yu Huang, Chin-Jung Wang, Yung-Kuei Soong, Hsin-Shih Wang, Mei Li Wang, Chieh Yu Lin, Chia Lin Chang

**Affiliations:** 1 Department of Obstetrics and Gynecology, Chang Gung Memorial Hospital Linkou Medical Center, Chang Gung University, 5 Fu-Shin Street, Kweishan, Taoyuan, Taiwan

**Keywords:** hysteroscopy, endometrium biopsy, IVF, repeated implantation failure, pregnancy

## Abstract

**Background:**

To test whether a site-specific hysteroscopic biopsy-induced injury in the endometrium during the controlled ovarian hyperstimulation cycle improves subsequent embryo implantation in patients with repeated implantation failure, a total of 30 patients who have had good responses to controlled ovulation stimulation but have failed to achieve pregnancy after two or more transfers of good-quality embryos were recruited in this prospective study.

**Methods:**

A single, site-specific hysteroscopic biopsy-induced injury was generated on the posterior endometrium at midline 10-15 mm from the fundus during the D4-D7 period of the ongoing controlled ovarian hyperstimulation cycle in six patients.

**Results:**

Patients received endometrial biopsy protocol achieved a pregnancy rate of 100%. By contrast, only 46% of patients with similar clinical characteristics (*N *= 24) achieved pregnancy without the hysteroscopic biopsy-induced endometrium injury (*p *< 0.05).

**Conclusions:**

Our proof-of-concept study demonstrates that a site-specific hysteroscopic endometrium injury performed during the ongoing *in vitro *fertilization (IVF) cycle, instead of injuries received during prior cycles, significantly improves clinical outcomes in patients with repeated implantation failure.

## Background

In assisted reproductive technology, procedures for culturing and transferring embryos have been continually improved over the last two decades. Yet the clinical pregnancy rate has not substantially improved over the last ten years (currently only 32.4~33.0% per IVF transfer as reported by ESHRE in 2010)[1], and many patients have suffered repeated implantation failure even in the most successful *in vitro *fertilization (IVF) clinics. Although no practical solutions for repeated implantation failure have emerged, an improved ability to control the endometrial environment for implantation promises to have a significant, positive impact on IVF outcomes.

Among the various potential causes of repeated implantation failure, uterine factors (e.g., thin endometrium, poor endometrial receptivity, and immunological incompatibility) have received the most attention in recent years [2]. It has been shown that endometrial receptivity could be modulated by a multitude of signaling molecules, including prostaglandins [3], growth factors, cytokines, chemokines, integrins, leukemia inhibitory factor [4,5], Wnt family ligands [6], and E-cadherin [7]. Whereas dysregulation of some of these factors could be associated with repeated implantation failure, key molecular mechanisms that underlie the regulation of endometrial receptivity remain to be elucidated [8].

Interestingly, earlier studies have shown that prior incidences of hysteroscopic endometrium biopsy are associated with increased rates of implantation, clinical pregnancy, and live birth among women who experienced repeated implantation failure but without obvious endometrium defects, suggesting that a hysteroscopic procedure in the nonconceptual cycle itself could be beneficial for improving pregnancy in subsequent IVF cycles [2,9-13]. This hypothesis has been supported--directly or indirectly--in a number of clinical settings [9-13]. Although earlier studies aiming to understand the effect of hysteroscopic endometrium biopsy on implantation have been methodologically diverse, a common denominator appears to have been the presence of local injuries prior to an IVF cycle. Because local inflammatory and angiogenesis reactions are indispensable for a successful implantation [14], an endometrial biopsy-induced injury could produce just such a local inflammatory and angiogenic environment between the endometrium and the conceptus, which, in turn, facilitates embryo implantation and subsequent pregnancy in earlier studies [9,15,16].

However, due to substantial variations in patient selection, timing, number and extent of endometrial injury applied, and techniques in earlier studies [9,10,17-19], the merits of endometrial biopsy injury on clinical outcomes in IVF clinics remain controversial [2]. In the present study, we reasoned that, if a local injury is the key causal agent for improved pregnancy outcomes, then a single site-specific mechanical injury performed shortly before a scheduled embryo transfer should yield a similar result. Herein, we demonstrate that a standard IVF protocol in which a minimal intervention in the form of a site-specific endometrial injury (which was not intended for diagnostic or operative purposes) during the controlled ovarian hyperstimulation cycle leads to a dramatic improvement in the pregnancy rate of patients with repeated implantation failure.

## Methods

### Subjects

The study was approved by the institutional ethics committee review board of Chang Gung Memorial Hospital Linkou Medical Center. Institutional Review Board approval was obtained in accordance with the Helsinki Declaration of 1975 on human experimentation. All patients gave written informed consent to participate in the study. Repeated implantation failure was defined as the failure to conceive following at least two cycles of IVF and the transfer of good-quality embryos. Select patients who have consent to receive the hysteroscopic procedure during the ongoing hyperstimulation cycle were recruited for the site-directed hysteroscopic biopsy treatment.

### Hyperstimulation protocol and hysteroscopic procedure

Ovulation induction was performed by the administration of recombinant FSH (175 - 225 IU/day, Puregon, N.V. Organon, The Netherlands) starting on day 2 of the menstrual cycle. GnRH antagonist (0.125 mg/day, Cetrorelix, Serono) was given beginning on day 5 of rFSH injection, or when the leading follicle reached 10 mm, to the day of hCG administration.

The single site-specific hysteroscopic biopsy procedure was performed using an electronic gynecological examination chair with the patient in a semirecumbent position. No premedication or local anesthetic was used. A Cuscoe's speculum was inserted into the vagina in order to visualize the cervix. Panoramic hysteroscopy was performed using a 4.9 mm diameter flexible hysteroscope (HYF-1T, Olympus Corporation, Shinjuku-ku, Tokyo, Japan). A 5% Dextrose distending medium was propelled by an electronic pump (Endomat, Kar Storz, Tuttlingen, Germany) with an intrauterine pressure of approximately 45 mm Hg. A claw forceps (A4033, Olympus Corporation, Shinjuku-ku, Tokyo, Japan) was introduced through a 2.2 mm working channel, and used to generate a local injury on the posterior endometrium at midline 10-15 mm from the fundus on D4 to D7 of the stimulation cycle. The depth and width of the injured site was 2 × 2 mm (i.e., a bite of the claw forceps). No antibiotic or hemostatic drug was administered after the procedure.

Once the leading follicle reached a size of at least 16 mm, 10, 000 IU of urinary hCG (Pregnyl 5, 000 IU, N.V. Organon, The Netherlands) was administrated to trigger ovulation. Ovum retrieval was performed 34 hr after the hCG injection. Embryos were transferred transvaginally on day 3 of culture or at the blastocyst stage, and the tip of the catheter (Labotect Gmbh, Germany) was placed at 10-15 mm from the uterine fundus with a transfer volume of ~10 μl. The luteal phase was supported with progesterone (8% vaginal-gel, Crinone, Prochieve). Clinical pregnancy was defined as visualization of fetal cardiac activity on transvaginal ultrasound examination.

### Statistical analysis

Results were presented as mean ± SD and analyzed by the Student's paired *t*-test. A *p *value < 0.05 was considered statistically significant.

## Results

**Figure 1:**
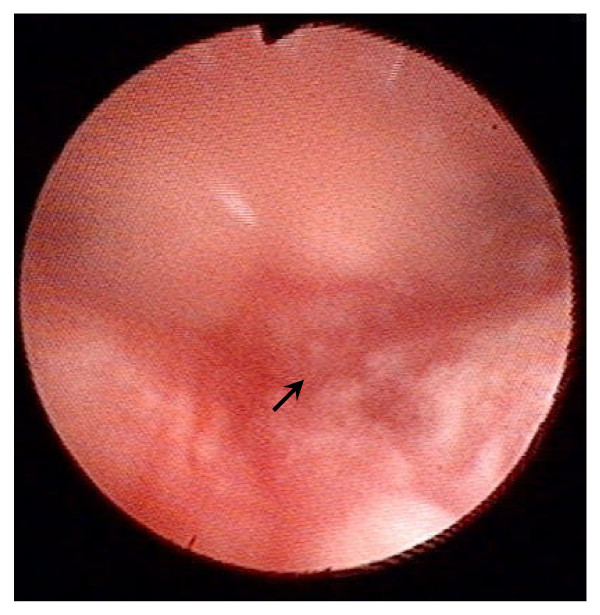
**Image of an operation during which an injury in the endometrium was generated using a flexible hysterofibrescope**. The injured site is indicated by an arrow.

During a 6-month period (March to October 2010), a total of 30 patients with predominant diagnoses of tubal factor (*n *= 7) or unexplained infertility (*n *= 23) were recruited. From this group, 6 patients who had consent to the procedure were subsequently treated with the hysteroscopic biopsy protocol. Two of these patients had predominant diagnoses of tubal factor, and four had unexplained infertility. These participants ranged in age from 31 to 39 (mean = 34.0 ± 3.0), and the mean number of previous IVF cycle attempts for these cases was 2.8 ± 0.8. In these patients, there was no difference in total dose of gonadotropin used, E_2 _levels on the day of hCG administration, fertilization rate, and the number of embryos transferred between previous unsuccessful cycles and the cycle during which the hysteroscopic biopsy-induced endometrium injury was performed (Figure 1, Table 1). In these patients, there was a trend toward having a thicker endometrium in cycles undergoing endometrial biopsy; however, the difference did not reach statistical significance (10.3 ± 2.0 mm vs. 8.7 ± 2.2 mm, *p *= 0.26). In addition, we observed a thin hypoechogenic endometrium on the day of endometrial biopsy in all patients, and a triple-line multi-layered pattern of endometrium was found on the day of hCG administration. The hysteroscopic technique obviously did not alter this expected transition in endometrial lining pattern.

**Table 1:** Clinical characteristics of patients who received a site-specific endometrium injury during the final IVF cycle

Characteristics	The IVF cycle with a biopsy	**Previous failed cycles**^**a**^	***p *value**^**b**^
Length of stimulation (day)	8.7 ± 1.0	9.9 ± 1.3	0.11
Total units of gonadotropin administered	1570 ± 377	1893 ± 270	0.10
Endometrium thickness on the day of hCG administration (mm)	10.3 ± 2.0	8.7 ± 2.2	0.26
E2 levels on the day of hCG administration (pg/mL)	1623 ± 550	1825 ± 879	0.64
Number of oocytes retrieved	11.3 ± 2.9	7.1 ± 3.1	0.03
Fertilization rate (%)	59 ± 29	49 ± 18	0.52
Number of embryos transferred	2.7 ± 1.2	2.3 ± 1.1	0.91
Implantation rate (sac number/transferred embryo number)	56%	0	
Pregnancy rate (clinical pregnancy/per ET)	100%	0	

^a^Measurements are the mean of parameters recorded in previous failed cycles

^b^Student's *t *-test of comparisons between the ongoing and prior IVF cycles

Of importance, we found that the implantation rate of patients in the treatment group was 56% (Tables 1 and 2), and a total of six pregnancies (100%)--four singleton, one twin, and one triplet--were recorded after the hysteroscopic intervention cycles. By contrast, 24 patients who had similar clinical characteristics but did not receive the hysteroscopic intervention recorded only a 21% rate of implantation per transferred embryo and a 46% (11/24) clinical pregnancy rate (Table 2). Consistently, the control group has a higher early abortion rate (27% (3/11)) as compared to the treatment group (0% (0/6)) at the 12^th ^week of gestation (Additional file 1, Supplemental Table S1).

**Table 2:** Comparison of clinical characteristics and treatment outcomes of patients with or without a site-specific hysteroscopic biopsy-induced endometrium injury during the final IVF cycle

Characteristics	Patients with a site-specific endometrium injury (N = 6)	Control patients (N = 24)	***p *value**^**a**^
Mean age (yr)	34 ± 3.0	35 ± 4.1	0.58
Weight (kg)	53.4 ± 3.0	54.3 ± 7.4	0.80
FSH (mIU/mL)	6.7 ± 2.3	6.9 ± 2.8	0.86
LH (mIU/mL)	4.2 ± 1.9	4.3 ± 2.1	0.89
Duration of Infertility (yr)	5.7 ± 2.8	6.4 ± 2.9	0.58
Total number of previously transferred embryos	5.0 ± 2.2	7.8 ± 3.0	0.06
Total number of previously IVF/ICSI cycles	2.8 ± 0.8	2.8 ± 0.9	0.88
Length of stimulation (day)	8.7 ± 1.0	9.2 ± 1.1	0.24
Total units of gonadotropin administrated (IU)	1570 ± 377	2206 ± 513	0.01
Endometrium thickness on the day of hCG administration (mm)	10.3 ± 2.0	9.8 ± 2.1	0.62
E2 levels on the day of hCG administration (pg/mL)	1623 ± 550	1822 ± 1078	0.67
Number of oocytes retrieved	11.3 ± 2.9	13.5 ± 7.4	0.50
Number of fertilizations	7.2 ± 4.6	6.3 ± 4.0	0.66
Fertilization rate (%)	59 ± 29	50 ± 22	0.45
Number of all embryos	3.8 ± 2.3	4.8 ± 2.0	0.32
Number of embryos transferred	2.7 ± 1.2	3.0 ± 0.5	0.29
Implantation rate (sac number/transferred embryo)	56%	21%	**0.01**
Pregnancy rate (clinical pregnancy/per ET)	100%	46%	**0.04**
Early abortion rate (abortion before 12 wks/total pregnancy)	0% (0/6)	27% (3/11)	

^a^Student's *t *-test of comparisons between control and treatment groups

## Discussion

Although major advances have been made in improving oocyte and embryo development in IVF clinics in the last two decades, hurdles remain in achieving high embryo implantation rates for many patients. For both patients and clinicians, one of the most discouraging issues in IVF treatments is the recognition of repeated implantation failure after transfers of good-quality embryos because it has been generally accepted that pregnancy and implantation rates are significantly lower for patients who undergo their second or third cycles of treatment as compared to those undergoing their first cycle of IVF [20].

Whereas causes of defective implantation in patients with repeated implantation failure could be multi-factorial or even originate from embryonic defects (e.g., genetic abnormalities and embryonic aneuploidy), earlier observations noted that patients who had received hysteroscopic biopsies prior to subsequent IVF cycle for various reasons had a higher pregnancy rate [2]. This observation has led to the hypothesis that endometrium injury might improve pregnancy in patients with repeated implantation failure as a result of subsequent inflammatory responses and changes in cytokine production in the endometrium [9-11,21]. Although a number of subsequent studies have reported significant correlations between incidence of hysteroscopic biopsy and improved pregnancy; however, it has not been directly defined what injury per se is the causal agent for the observed improvement because almost all earlier studies have applied blind and nonspecific injuries during cycles prior to the final controlled ovarian hyperstimulation cycle. In addition to variations in the type and extent of injuries received by patients, it is conceivable that the effect of endometrium injuries given long before the subsequent embryo-transferring cycle could be lessened by subsequent menses.

Because the exact injury and timing required to effectively improve implantation is not clear, the value of hysteroscopic injury for improving pregnancy in patients with repeated implantation failure remains to be established. To specifically investigate whether a local injury itself is sufficient to improve implantation, we applied a hysteroscopic biopsy at a specific site of the endometrium (posterior wall 10-15 mm from the fundus) during a specific time window (D4 to D7) of the ongoing stimulation cycle--instead of relying on nonspecific injuries from prior cycles. This design has effectively eliminated many confounding effects associated with the extent and duration of nonspecific injuries administered in earlier studies. In addition, by incorporating the intervention process into the traditional IVF procedure, this study has allowed us to compare differences not only between treatment groups but also between the biopsy cycle and earlier failed cycles in these patients. Furthermore, we chose to perform the biopsy at a specific location of the endometrium because it has been recently shown that the deposition of embryos at 10 to 20 mm from the uterine fundus allows patients receiving IVF to reach a higher pregnancy rate [22]. Consistent with our hypothesis, the results demonstrated that a site-specific hysteroscopic biopsy-induced endometrium injury during the follicular phase of an ongoing IVF cycle can significantly improve implantation and pregnancy rates. This result is important because this hysteroscopic intervention protocol could become a standard procedure for treating repeated implantation failure. Nonetheless, it is important to note that our proof-of-concept study was performed with a small group of patients, and that there are differences in the total units of gonadotropin administered and the total number of previously transferred embryos between the two study groups (Table 2). Future studies with larger populations of patients are needed to validate current findings.

For a successful pregnancy in placental mammals, two major physiological events have to occur: (a) building a receptive uterine endometrium for embryo implantation, and (b) protecting the semiallotypic fetus from attacks by the maternal immune system. Although the exact mechanism by which the local injury improves pregnancy remains to be investigated, our study has strengthened the hypothesis that a mechanical injury may enhance uterine receptivity. By provoking the immune system with an injury, the immune response and inflammatory reactions associated with wound healing in the endometrium may, in turn, increase the endometrial receptivity to the semiallotypic embryo [23]. Alternatively, the local injury could generate a focus for the accumulation of uterine dendritic cells and accompanying increases in innate immune molecules [24], or provide an enhanced angiogenic environment enriched with cytokines and growth factors known to be essential for normal trophoblast invasion.

## Conclusions

As a whole, our pilot study has provided direct evidence to support the hypothesis that a local injury performed during an ongoing IVF cycle is beneficial for implantation in patients with repeated implantation failure, and a discrete procedure for further testing the hypothesis that a site-specific mechanical injury could improve pregnancy outcomes. In addition, we envision that the establishment of this discrete procedure could provide a platform for systematic investigations of molecular mechanisms underlying endometrium injury-mediated improvement in implantation and the role of various inflammatory factors in regulating endometrium receptivity in humans.

## List of abbreviations

IVF: *in vitro *fertilization; FSH: follicle stimulating hormone; hCG: human chorionic gonadotropin.

## Competing interests

The authors declare that they have no competing interests.

## Authors' contributions

CLC contributed to conception, design, and initiation of the study. CJW, MLW, CYL, and CLC performed the clinical procedures. SYH made substantial contributions to the analysis of samples and data. CLC, YKS, and HSW contributed to the interpretation of the data and the preparation of the manuscript. All authors read and approved the final manuscript.

## Supplementary Material

Additional file 1 **Supplemental Table S1**. Clinical outcomes of patients with a clinical pregnancy. Click here for file
