# Supplementary material for: Site-specific endometrial injury improves implantation and pregnancy in patients with repeated implantation failures
Source: Reprod Biol Endocrinol. 2011 Oct 21;9:140. doi: 10.1186/1477-7827-9-140 (PMC3210086; doi:10.1186/1477-7827-9-140)
Supplement: Additional file 1 — Supplemental Table S1. Clinical outcomes of patients with a clinical pregnancy. [file 1477-7827-9-140-S1.PDF]

Supplementary Table 1. Clinical outcomes of patients with a clinical pregnancy.

**Patients who received a hysteroscopic biopsy-induced endometrium injury (N=6)**				
Case No.	Gestational age (wks)	Delivery mode	Estimated fetal BW (g)	BW at birth (g)/ sex
1	34	C-section		1995/F; 1978/F
2	39	delivered		3335/F
3	40	C-section		2930/M
4	39	delivered		2800/F
5	37	delivered		2750/F
6	33	ongoing	1650/F;1950/M	
**Control patients who achieved clincal pregnancy (N=11)**				
Case No.	Gestational age (wks)	Delivery mode	Estimated fetal BW (g)	BW at birth (g)/ sex
1	39	delivered		3620/M
2	34	delivered		2045/F; 2160/F
3	—	lost follow-up at 14-wks		
4	—	lost follow-up at 13-wks		
5	—	**aborted at 8-wks**		
6	—	lost follow-up at 12-wks		
7	—	**aborted at 5-wks**		
8	39	delivered		2915/M
9	36	C-section		2945/M; 2775/M
10	39	delivered		3700/F
11	—	**aborted at 6-wks**		
